# Discovery of piperlongumine as a potential novel lead for the development of senolytic agents

**DOI:** 10.18632/aging.101100

**Published:** 2016-11-19

**Authors:** Yingying Wang, Jianhui Chang, Xingui Liu, Xuan Zhang, Suping Zhang, Xin Zhang, Daohong Zhou, Guangrong Zheng

**Affiliations:** ^1^ Department of Pharmaceutical Sciences, College of Pharmacy, University of Arkansas for Medical Sciences, Little Rock, AR 72205, USA; ^2^ Winthrop P. Rockefeller Cancer Institute, University of Arkansas for Medical Sciences, Little Rock, AR 72205, USA

**Keywords:** piperlongumine, aging, senescent cells, senolytic agents, ABT-263, reactive oxygen species, synergistic effect

## Abstract

Accumulating evidence indicates that senescent cells play an important role in many age-associated diseases. The pharmacological depletion of senescent cells (SCs) with a “senolytic agent”, a small molecule that selectively kills SCs, is a potential novel therapeutic approach for these diseases. Recently, we discovered ABT-263, a potent and highly selective senolytic agent, by screening a library of rationally-selected compounds. With this screening approach, we also identified a second senolytic agent called piperlongumine (PL). PL is a natural product that is reported to have many pharmacological effects, including anti-tumor activity. We show here that PL preferentially killed senescent human WI-38 fibroblasts when senescence was induced by ionizing radiation, replicative exhaustion, or ectopic expression of the oncogene *Ras*. PL killed SCs by inducing apoptosis, and this process did not require the induction of reactive oxygen species. In addition, we found that PL synergistically killed SCs in combination with ABT-263, and initial structural modifications to PL identified analogs with improved potency and/or selectivity in inducing SC death. Overall, our studies demonstrate that PL is a novel lead for developing senolytic agents.

## INTRODUCTION

Cellular senescence, an essentially irreversible arrest of cell proliferation, can be triggered when cells experience a potential risk for malignant transformation due to the activation of oncogenes and/or DNA damage [1–7]. While eliminating aged or damaged cells by inducing senescence is an effective barrier to tumorigenesis, the accumulation of senescent cells (SCs) over time compromises normal tissue function and contributes to aging and the development of age-associated diseases [6, 8, 9]. Often, SCs secrete a broad spectrum of pro-inflammatory cytokines, chemokines, growth factors, and extracellular matrix proteases, a feature collectively termed the senescence-associated secretory phenotype. These factors degrade the local tissue environment and induce inflammation in various tissues and organs if SCs are not effectively cleared by immune system [6, 8–11].

Studies have shown that the genetic clearance of SCs extends the lifespan of mice and delays the onset of several age-associated diseases in both progeroid and naturally-aged mice [12–15]. It has also been shown that rapamycin and metformin increase lifespan in mice and marmoset monkeys, by suppressing the induction of senescence [16–20]. These findings support the hypothesis that SCs play a causative role in aging and age-associated diseases [6, 21, 22] and, importantly, highlight the tremendous therapeutic potential of pharmacologically targeting SCs [23, 24]. Consistent with these findings, we have shown that ABT-263 (navitoclax), an inhibitor of the antiapoptotic Bcl-2 family proteins, acts as a potent senolytic agent to deplete SCs *in vivo* and functionally rejuvenates hematopoietic stem cells in both sublethally irradiated and naturally-aged mice [25]. Complementary studies from other labs have confirmed that the Bcl-2 protein family is a promising molecular target for the development of senolytic drugs [26, 27]. These studies further establish the concept that the pharmacological depletion of SCs is a promising, novel approach for treating age-associated diseases [28].

**Figure 1:**
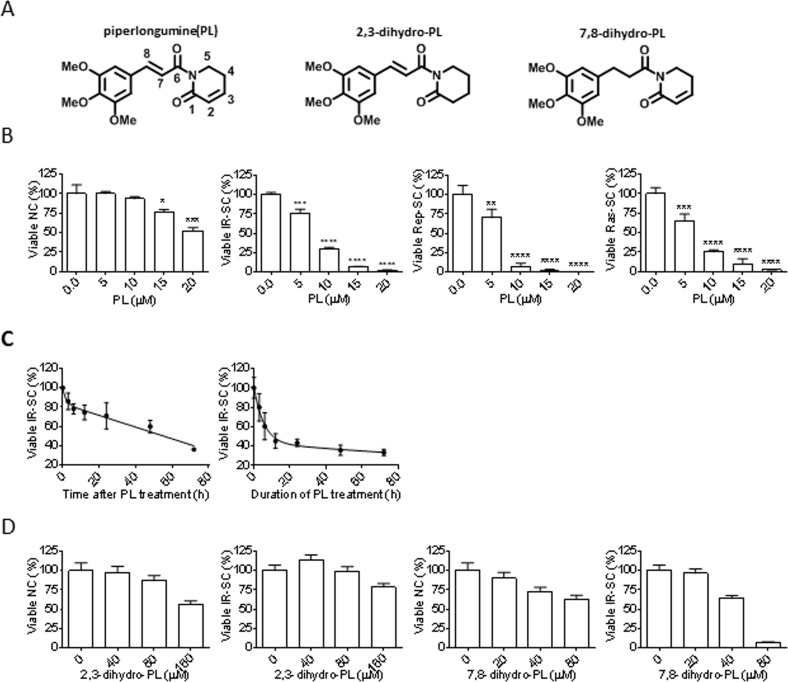
Senolytic activity of piperlongumine (PL) (**A**) Structures of PL, 2,3-dihydro-PL, and 7,8-dihydro-PL. (**B**) Quantification of viable WI-38 non-senescent cells (NC), IR-induced senescent cells (IR-SC), replication-exhausted senescent cells (Rep-SC), or Ras-induced senescent cells (Ras-SC) 72 h after treatment with increasing concentrations of PL (*n* = 3). (**C**) Quantification of viable IR-SCs over time after treatment with 10 μM PL (left) or after incubation with 10 μM PL, removal of the drug, and further culture for 72 h (right) (*n* = 3). (**D**) Quantification of viable WI-38 NCs and IR-SCs 72 h after treatment with increasing concentrations of 2,3-dihydro-PL or 7,8-dihydro-PL (*n* = 3). Data are represented as the mean ± SEM.

ABT-263 was identified by screening a small library of structurally diverse, rationally-selected small molecules that target pathways predicted to be important for SC survival [25]. By titrating their cytotoxicity against normal human WI-38 fibroblasts and ionizing radiation (IR)-induced senescent WI-38 fibroblasts, this targeted screen also identified the promising senolytic agent piperlongumine (PL, Fig. 1A); PL is a natural product isolated from a variety of species in the genus Piper [29]. Here, we report the characterization of PL as a potential novel lead for the development of senolytic agents.

## RESULTS

### Piperlongumine is a potential senolytic agent

Because we identified PL as a potential senolytic agent by screening a library of rationally-selected compounds with IR-induced senescent WI-38 fibroblasts, we tested its ability to selectively kill senescent human WI-38 fibroblasts induced by different means. PL exhibited moderate selectivity in reducing the viability of IR-induced WI-38 SCs (IR-SCs) compared to non-senescent WI-38 cells (NCs) (Fig. 1B and Table 1), and PL induced cell death in a time-dependent manner (Fig. 1C). We also assessed the survival of WI-38 cells in which senescence was induced by replicative exhaustion or by ectopic expression of the oncogene *Ras* (Fig. 1B). Replicative WI-38 SCs, which were previously shown to be more resistant to ABT-263 [25], were slightly more sensitive to PL (Fig. 1B) than IR- and Ras-induced SCs. The mechanisms underlying the difference of SCs induced by different stimuli have yet to be elucidated.

**Table 1:** EC_50_ values and selectivity of PL in WI-38 cells

Cell types	EC_50_ (μM)	EC_50_ Ratio (NC/SC)
NC	20.28	-
IR-SC	7.97	2.54
Rep-SC	6.24	3.25
Ras-SC	7.09	2.86

Structurally, PL contains two electrophiles, the C2-C3 and C7-C8 α,β-unsaturated imides, both of which are important for the toxicity of PL in cancer cells [30]. Thus, we investigated whether the integrity of the two-electrophile system was also important for the ability of PL to kill SCs. Consistent with the findings in cancer cells, 2,3-dihydro-PL and 7,8-dihydro-PL (Figure 1A), in which the C2-C3 olefin or the C7-C8 olefin was saturated, respectively, showed little or no senolytic activity toward IR-SCs (Fig. 1D).

### Piperlongumine induces apoptosis in SCs

**Figure 2:**
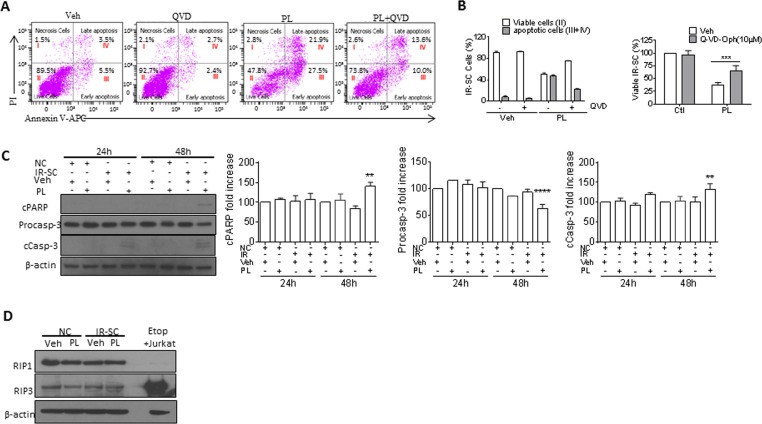
PL kills SCs by apoptosis (**A**) Representative flow cytometric plots to measure apoptotic WI-38 IR-SCs at 48 h after treatment with vehicle (Veh), 10 μM PL, 10 μM Q-VD-Oph (QVD), or the combination of PL and QVD. (**B**) Quantification of the percentage of viable (gate II: PI^−^ Annexin V^−^) and apoptotic (gates III and IV: PI^−^ Annexin V^+^ and PI^+^ Annexin V^+^) (right) IR-SCs 48 h after treatment as in (**A**) (left), and quantification of the percentage of viable IR-SCs 72 h after treatment as in (**A**) (right). (**C**) Representative western blot and quantitative analysis of cleaved-poly(ADP-ribose) polymerase (cPARP), procaspase-3 (Procasp-3), cleaved caspase-3 (cCasp-3), and β-actin in NCs and WI-38 IR-SCs 24 h and 48 h after incubation with Veh or 10 μM PL. (**D**) Representative western blot analysis of RIP1, RIP3, and β-actin in WI-38 NCs and IR-SCs 24 h after incubation with Veh or 10 μM PL. A cell lysate of etoposide-treated Jurkat cells was used as a positive control. Data are represented as the mean ± SEM.

Next, we investigated the mechanism by which PL selectively kills SCs. Because PL induces apoptosis in cancer cells [31–41], we hypothesized that the same is true for SCs. We used Annexin V and propidium iodide staining and subsequent fluorescence-activated cell sorting to detect apoptosis, respectively, in senescent WI-38 cells. PL treatment increased the number of Annexin-V-positive cells in SCs by 5.5-fold when compared to the vehicle group (Fig. 2A). To further confirm that PL killed cells by apoptosis, we treated IR-induced WI-38 SCs with the pan-caspase inhibitor Q-VD-OPh (QVD) [42] to inhibit apoptosis. Ten μM QVD, in the presence of PL, significantly reduced apoptosis and partially rescued SCs from PL-induced death (Fig. 2A, B). In addition, western blot analysis showed elevated levels of activated caspase-3 and degradation of poly(ADP-ribose) polymerase (PARP) in PL-treated IR-SCs (Fig. 2C), confirming the apoptotic cell-death mechanism. Furthermore, PL had no effect on the levels of receptor-interacting protein kinase 1 and 3 (RIP1 and RIP3), indicating that PL did not induce necroptosis in IR-SCs (Fig. 2D) [43].

### Piperlongumine kills senescent cells through an ROS-independent mechanism

**Figure 3:**
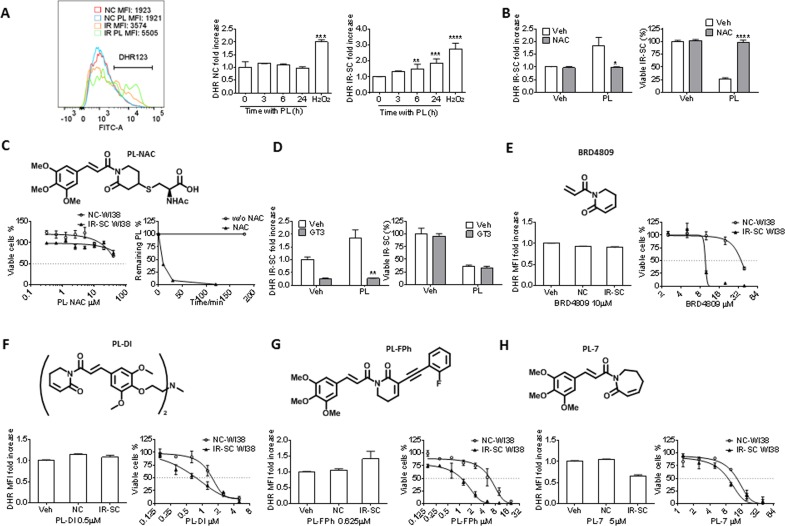
Effect of PL and its analogs on ROS production and senolytic activity in WI-38 IR-SCs (**A**) Representative flow cytometric analysis of ROS production in NCs and IR-SCs 24 h after incubation with or without PL by DHR (left) (MFI, mean fluorescence intensity) and quantification of the fold increase of ROS levels in WI-38 NCs and WI-38 IR-SCs cells at the indicated times (middle and right) after incubation with 10 μM PL. As a positive control, cells were treated with 100 μM of H2O2 for 2 h, the H2O2 was removed, and cells were cultured for an additional 24 h (*n* = 3). (**B**) Quantification of the fold increase in DHR-123 MFI (left) in WI-38 IR-SCs 24 h after treatment with Veh, 10 μM PL, 2 mM NAC (pretreatment overnight), or the combination of PL and NAC, and (right) the percentage of viable WI-38 IR-SCs 72 h after treatment with Veh, 10 μM PL, 2 mM NAC (pretreatment overnight), or the combination of PL and NAC (*n* = 3). (**C**) Structure of PL-NAC and (Left) quantification of viable WI-38 NCs and WI-38 IR-SCs 72 h after treatment with increasing concentrations of PL-NAC (*n* = 3). (Right) Percentage of 10 μm PL remaining in the culture medium vs. time with or without 2mM NAC. (**D**) Left panel: quantification of the fold increase in DHR MFI (left) of WI-38 IR-SCs 24 h after treatment with Veh, 10 μM PL, 5 μM γ-tocotrienol (GT3, pretreatment overnight), or the combination of PL and GT3; and right panel: the percentage of viable WI-38 IR-SCs 72 h after treatment with Veh, 10 μM PL, 5 μM GT3 (pretreatment overnight), or the combination of PL and GT3 (*n* = 3). (**E-H**) Quantification of the fold increase in DHR-123 MFI after 24 h treatment (left) and viability of WI-38 NCs and WI-38 IR-SCs 72 h treatment (right) after they were treated with increasing concentrations or (**E**)10 μM BRD4809, (**F**) 0.5 μM PL-DI, (**G**) 0.625 μM PL-FPh, and (**H**) 5 μM PL-7 (*n* = 3). Data are represented as the mean ± SEM.

Initially, PL has been proposed to selectively induce cancer cell death by increasing reactive oxygen species (ROS) production, based on the observation that PL elevates cellular ROS levels in various cancer cells, but not in normal cells [31]. However, structural modifications to PL have revealed that there is no correlation between a PL analog's ability to increase ROS and its toxicity toward cancer cells, leading to the conclusion that ROS-independent mechanisms are also involved in cancer cell death [30]. We hypothesized that the same scenario is true for the PL-induced killing of SCs. We used the non-fluorescent ROS indicator dihydrorhodamine 123 (DHR 123), which can passively diffuse across membranes where it is oxidized to green fluorescent rhodamine 123 in the presence of ROS, and flow cytometry to determine if PL increased ROS in IR-SCs. Treatment with 10 μM PL for 6 or 24 h significantly elevated ROS levels in IR-SCs compared to vehicle-treated IR-SCs or non-senescent WI-38 cells with PL treatment, whereas IR-SCs have a higher baseline level of ROS (Fig. 3A). In addition, similar to the results obtained in cancer cells [31, 33–35, 41, 44–47], co-treatment with 2 mM N-acetyl-L-cysteine (NAC), an antioxidant, fully reversed PL-induced ROS elevation and cell death (Fig. 3B), suggesting that the selective induction of ROS in SCs may be the basis for the senolytic activity of PL. However, a number of small molecules, including hydrogen peroxide, part-henolide, arsenic trioxide, phenethyl isothiocyanate, auranofin [a thioredoxin reductase inhibitor], buthionine sulfoximine [a γ-glutamylcysteine synthetase inhibitor], and decyl-triphenylphosphonium, that were previously shown to kill cancer cells by inducing oxidative stress were not able to selectively kill IR-SCs [25]. This finding suggests that ROS elevation alone is insufficient to selectively kill SCs. Interestingly, it has been reported that PL can chemically react with the sulfhydryl group of methyl thioglycolate to form the product of conjugate addition at C3 [30]. Based on this, we hypothesized that NAC, rather than acting as an ROS scavenger, inactivates PL through a similar reaction in cell culture media. In support of this hypothesis, we observed that PL (10 μM) disappeared within 2 h after co-incubation with NAC (2 mM) in cell culture media under conditions mimicking a cell viability assay (Fig. 3C), forming the corresponding hetero-conjugated product, NAC-PL (Fig. 3C). NAC-PL was isolated from this reaction, and it exhibited diminished toxicity toward IR-SCs (Fig. 3C). To further investigate the role of ROS in PL-induced SC death, we treated IR-SCs with PL in the presence of a different, potent antioxidant, γ-tocotrienol (GT3, 5 μM) [48]. GT3 did not decrease PL-induced cell death in IR-SCs, although GT3 reversed the PL-induced increase in ROS (Fig. 3D). These data suggest that ROS were not involved in the SC death induced by PL.

To determine if PL-based senolytic agents with increased potency/selectivity could be developed, we synthesized a series of PL analogs that have been reported as potent anti-cancer agents. No obvious correlation between ROS induction and senolytic potency was observed in these analogs. Specifically, BRD4809 [49], an abbreviated PL analog (Fig. 3E), and PL-DI [30], a PL dimer (Fig. 3F), showed unchanged or increased potency against IR-SCs, respectively, compared to PL; however, these analogs did not affect ROS levels in SCs at concentrations near their EC_50_ values for SC viability (Table 2). On the other hand, PL-FPh [30], which contains an alkenyl substituent at C2 of PL, selectively induced ROS production in IR-SCs and had increased potency and selectivity in killing these SCs when compared to PL (Fig. 3G). Finally, PL-7 [30], a PL analog with an enlarged ring, inhibited ROS production, yet retained the senolytic potency of PL toward IR-SCs (Fig. 3H). Taken together, these results further confirm that PL and its analogs kill SCs in an ROS-independent manner.

**Table 2:** EC_50_ values and selectivity of PL analogs in WI-38 cells

PL analogs	EC_50_ (μM)		EC_50_ ratio (NC/IR)
	NC	IR	
BRD4809	35.7	9.7	3.68
PL-DI	1.53	0.76	2.01
PL-7	12.96	8.85	1.46
PL-FPh	5.87	1.11	5.29

### The synergistic senolytic effect of piperlongumine and ABT-263

**Figure 4:**
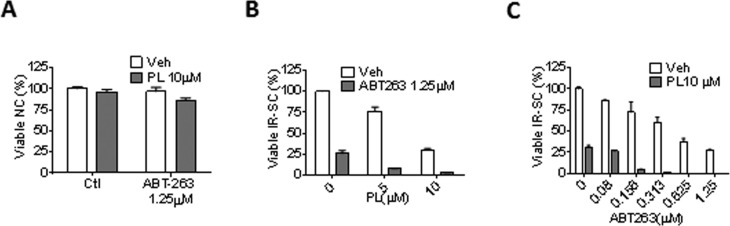
PL synergistically and selectively kills SCs in combination with ABT-263 (**A**) Quantification of NC viability 72 h after the cells incubation with vehicle, 1.25 μM ABT-263, 10 μM PL, or the combination of ABT-263 and PL (*n* = 3). (**B**) Quantification of WI-38 IR-SC viability 72 h after incubation with vehicle, 1.25 μM ABT-263, 5 or 10 μM PL, or the combination of ABT-263 and PL (*n* = 3-5). (**C**) Quantification of WI-38 IR-SC viability 72 h after incubation with vehicle, 10 μM PL, 0.08-1.25 μM ABT-263, or the combination of ABT-263 and PL (*n* = 3-6). Data are represented as the mean ± SEM.

PL has been tested for its synergistic anti-tumor effect in combination with TNF-related apoptosis-inducing ligand [46], ataxia telangiectasia and Rad3-related protein inhibition [50], or a chemotherapeutic agent, such as cisplatin [33, 34], paclitaxel [34], docetaxel [51], and gemcitabine [39]. Thus, we investigated the synergistic senolytic effect of PL and ABT-263 on IR-SCs. We tested 1.25 μM ABT-263 with 5 or 10 μM PL and 10 μM PL with 0.08-1.25 μM ABT-263; the ABT-263 concentrations were selected based on our recent studies [25]. The combination of 10 μM PL with 1.25 μM ABT-263 did not induce significant toxicity in non-senescent WI-38 cells (Fig. 4A). When the combinations were applied to IR-SCs, however, we observed significant synergistic effects (Fig. 4B-C). For example, treatment of SCs with 10 μM PL or 1.25 μM ABT-263 individually resulted in cell viability of 30.4% and 25.8%, respectively. However, the combined treatment with PL and ABT-263 killed almost all IR-SCs (Fig. 4C). The coefficient of drug interaction (CDI) method [52] was then used to evaluate the effects of PL and ABT-263; Table 3 gives the CDI values for these combinations. An additive effect for the combination of 10 μM PL and 0.08 μM ABT-263 (CDI = 0.99) was observed. The CDI values for the other combinations ranged from 0.02-0.41, indicating that PL and ABT-263 exerted a strong synergistic senolytic effect on IR-SCs. It is worth noting that, in our previous studies, increasing the concentration of ABT-263 from 1.25 μM to 5 μM did not increase cell killing in WI-38 SCs. PL appeared to eliminate the subpopulation of IR-SCs that was resistant to ABT-263.

**Table 3:** CDI values for the combination of PL and ABT-263

ABT-263 (μM)	PL (μM)	CDI
0.08	10	0.99
0.156	10	0.20
0.313	10	0.05
0.625	10	0.02
1.25	10	0.38
1.25	5	0.41

## DISCUSSION

Selective depletion of SCs is a potentially novel anti-aging strategy that may prevent cancer and various human diseases associated with aging and rejuvenate the body to live a longer, healthier life. As such, several senolytic agents, including ABT-263, have been identified recently [23, 25–27], demonstrating the feasibility of pharmacologically targeting SCs. However, ABT-263 induces thrombocytopenia [53], and it remains to be determined whether ABT-263 can be used to safely treat age-related diseases, since individuals may require long-term treatment with a senolytic drug. Thus, it is necessary to identify a safer senolytic drug. In the present study, we evaluated PL as a novel senolytic agent. PL induced caspase-mediated apoptosis in SCs and effectively killed SCs induced by IR, replicative exhaustion, or ectopic expression of the oncogene *Ras*. Similar to the observations in cancer cells versus normal cells, PL elevated ROS levels in IR-SCs, but not in non-senescent WI-38 cells.

Because NAC blocks PL-induced ROS elevation in cancer cells and abolishes the anti-tumor effect of PL, it has been proposed that induction of ROS production is a key mechanism of PL-induced cancer cell apoptosis [31, 33–35, 41, 45–47]. Indeed, we showed that co-treatment of IR-SCs with PL and NAC fully reversed the senolytic effect of PL. However, we found that PL was chemically inactivated by NAC in culture media through a conjugated addition reaction between the sulfhydryl group of NAC and the C2-C3 α,β-unsaturated imide group of PL; the resulting adduct, NAC-PL, was not senolytic. Based on these findings, caution is warranted when using NAC or similar compounds that contain a nucleophilic sulfhydryl group, such as dithiothreitol [54], as an ROS scavenger to study ROS inducers such as PL. In contrast, GT3, a potent ROS scavenger that does not react with PL, effectively blocked PL-induced ROS elevation but had no inhibitory effect on PL-induced SC death. In addition, through evaluation of PL analogs, we found that there is no correlation between senolytic potency and ROS-induction in IR-SCs. These results led us to conclude that the senolytic activity of PL is ROS independent.

Unlike ABT-263, the precise mechanism of action (MOA) by which PL induces SC apoptosis remains unclear. PL modulates the activity of many cell signaling and survival pathways in cancer cells [31], and a number of studies have investigated the MOA by which PL induces apoptosis in these cells [30, 35–40, 45, 54–69]. Data from these studies may be translatable to PL-induced SC apoptosis because SCs and cancer cells share some common pro-survival pathways [23]. In addition, mass spectrometry-based proteomic approaches using probes derived from PL could be used to identify direct molecular target(s) of PL in SCs. In this regard, novel anti-senescent protein target(s) and MOAs could be identified, making it possible to develop promising novel classes of senolytic agents. Importantly, PL appears to be safe; the maximum tolerated dose in mice is very high, and it maintains high bioavailability after oral administration [31]. Furthermore, our initial structural modifications to PL demonstrate that we can develop PL analogs with increased potency and selectivity toward SCs (Fig. 3), supporting the use of PL as a lead for further drug discovery and development.

Another potential use of PL and its derivatives is in combination with ABT-263, or other inhibitors of Bcl-2 family proteins, for a synergistic senolytic effect. Although ABT-263 is a highly specific senolytic agent, it causes transient thrombocytopenia and neutropenia in patients [70]; this results from its inhibitory effect on Bcl-xL, which is important for platelet survival [71, 72]. We showed that PL had a strong synergistic effect on the senolytic activity of ABT-263 *in vitro*, potentially reducing the dose of ABT-263 needed to effectively deplete SCs. We expect this therapeutic approach would significantly reduce ABT-263-induced thrombocytope-nia, making senolytic treatment with ABT-263 safer.

Although clearance of SCs with a senolytic drug may be used to treat some age-related diseases, it is well recognized that cellular senescence is also functionally linked to many beneficial physiological processes, such as wound healing, tissue remodeling, and embryonic development [73]. Attempt to clear SCs in certain situations may produce some side effects. Therefore, we should proceed with caution to use senolytic drugs to treat age-related diseases before we have a better understanding of their risks.

## MATERIALS AND METHODS

### Cells, induction of senescence, and senolytic agents

Human WI-38 fibroblasts (WI-38, catalog no. CCL-75, American Type Culture Collection, Manassas, VA) were cultured in a complete cell culture medium (CM) (Dulbecco's Modified Eagle Medium supplemented with 10% Fetal Bovine Serum, FBS; catalog no. 16000044, Thermo Fisher Scientific, Waltham, MA) supplemented with 100 U/ml penicillin and 100 μg/ml streptomycin (purchased from Atlanta Biologicals, Norcross, GA) in a 37°C, humidified incubator with 5% CO_2_.

Low-passage WI-38 (< 25 passages) cells were used as controls or for the induction of senescence.

#### Replicative senescence

To induce replicative senescence (Rep-SC), WI-38 cells were subcultured until they stopped dividing and became senescent (after approximately 38 passages for WI-38).

#### Ionizing radiation-induced senescence

To induce senescence with ionizing radiation (IR), WI-38 cells, roughly 70% confluent, were exposed to 15 Gy of IR in a J.L. Shepherd Model Mark I ^137^Cesium Ƴ-irradiator (J.L. Shepherd, Glendale, CA) at a dose rate of 1.080 Gy/min. Three days after irradiation, cells were passaged once at a 1:3 dilution. WI-38 cells became fully senescent 10 d after irradiation.

#### Ras-induced senescence

WI-38 cells were made senescent by ectopically expressing the oncogene *Ras* (Ras-SC), as previously described [25].

PL was purchased from Biovision (catalog no. 1919-10; Milpitas, CA). ABT-263 was purchased from Selleckchem (catalog no. S1001; Houston, TX). The PL analogs 2,3-dihydro-PL, 7,8-dihydro-PL, BRD4809, PL-DI, PL-FPh, and PL-7 were synthesized according to reported methods, with minor modifications [30, 49]. PL-NAC was obtained by incubating equal volume of 20 μM PL with 4 mM NAC in culture media at 37°C for 30 min, followed by extraction with methylene chloride and silica gel column purification. The structure of PL-NMR was characterized by NMR and MS: ^1^H NMR (400 MHz, CDCl_3_) δ 7.59 (d, *J* = 15.6 Hz, 1H), 7.28 (d, *J* = 15.6 Hz, 1H), 6.86 (br, 1H), 6.76 (s, 2H), 4.68 (s, 1H), 3.99 (m, 1H), 3.88–3.75 (m, 9H), 3.63 (m, 1H), 3.27 (m, 1H), 3.13–2.87 (m, 3H), 2.69–2.44 (m, 1H), 2.25 (m, 1H), 2.04 (s, 3H), 1.81 (m, 1H) ppm; ESI-MS *m/z* 479.2 [M-H]^+^.

### Cell viability assays

Cell viability was measured with flow cytometry, as previously described [25].

### Calculation of EC_50_ values

Dose-response curves were generated for each senolytic agent, and the half-maximal effective concentrations (EC_50_ values) were calculated with GraphPad Prism 6 software.

### ROS assay

Control, non-senescent WI-38 cells were plated in 24-well plates (60,000 cells/well). IR-induced WI-38 senescent cells (10 d after 15 Gy IR) were plated in 6-well plates (50,000 cells/well) and allowed to recover. Cells were incubated overnight with NAC (2 mM; catalog no. 138061, Sigma-Aldrich, St. Louis, MO) and GT3 (5 μM; isolated from annatto oil). The next day, the cells were treated with dilutions of compounds in DMSO and incubated for 24 h, or as indicated. The medium was then replaced with pre-warmed DMEM (no supplements) containing 1 μM dihydrorhodamine 123 (DHR 123, catalog no. D632, Thermo Fisher Scientific), and the cells were incubated at 37°C for 30 min. The cells were then harvested with trypsin and washed twice with PBS. Mean fluorescence intensity (MFI) was determined with a BD LSR II flow cytometer (BD Biosciences, San Jose, CA).

### Apoptosis assay

WI-38 cells were pretreated with vehicle or 10 μM Q-VD-Oph (QVD, catalog no. A1901, APExBIO, Houston, TX) for 4 h. Cells were then treated with 10 μM PL for the indicated time. The cells were harvested and washed twice with Annexin V binding buffer and then stained with Alexa Fluor 647-Annexin V (1: 50, catalog no. 640912, BioLegend, San Diego, CA) and propidium iodide (PI, 10 μg/ml, catalog no. P4170, Sigma-Aldrich), according to the manufacturer's instructions (Biotium, Hayward, CA). All of the stained cells were analyzed with the BD LSR II flow cytometer.

### Western blot analysis

Cells were lysed in RIPA buffer with EDTA and EGTA (catalog no. BP-115DG, Boston BioProducts, Ashland, MA), supplemented with 1% Phosphatase Inhibitor Cocktail 3 (catalog no. P0044, Sigma-Aldrich) and 1% Protease Inhibitor Cocktail (catalog no. P8340, Sigma-Aldrich). An equal amount of protein (15-30 μg/lane) from each cell extract was resolved on a 12% SDS-PAGE gel. Proteins were blotted to a NOVEX PVDF membrane (catalog no. LC2002, Life Technologies) by electrophoresis. The membranes were blocked with TBS-T blocking buffer (5% nonfat milk in 25 mM Tris-HCL, pH 7.4; 3 mM KCl; 140 mM NaCl; and 0.05% Tween) and probed with primary antibodies (at a predetermined optimal concentration) overnight at 4°C or for 1 h at room temperature. After extensive washing with TBS-T, the membranes were incubated with an appropriate peroxidase-conjugated secondary antibody (Jackson ImmunoResearch Europe, Suffolk, UK) for 1 h at room temperature. After three washes with TBS-T, the proteins of interest were detected with ECL Western Blotting Detection Reagents (catalog no. WBKLS0100, EMD Millipore, Newmarket, Suffolk, UK) and recorded with autoradiography (Pierce Biotech, Rockford, IL, USA). The primary antibodies included cleaved-Poly (ADP-ribose) polymerase (catalog no. 9541, Cell Signaling Technology, Boston, MA), Procaspase-3 (catalog no. 9662S, Cell Signaling Technology), cleaved-caspase 3 (catalog no. 9664S, Cell Signaling Technology), RIP1 (D94C12, catalog no. 3493S, Cell Signaling Technology), β-actin (catalog no. SC-1615, Santa Cruz Biotechnology, Dallas, TX), and RIP3 (catalog no. IMG-5846A, IMGENEX, San Diego, CA).

### Statistical analysis

The data displayed normal variance. The data were analyzed by analysis of variance (ANOVA) with Graphpad Prism from GraphPad Software (San Diego, CA). In the event that ANOVA justified post hoc comparisons between group means, the comparisons were made with Neuman-Keuls or Tukey's multiple-comparisons test. *P* < 0.05 was considered to be significant. CDI was calculated as: CDI = AB/(A × B). AB represents the percent of viable cells remaining after the treatment with the combined drugs, while A and B represent the percent of viable cells remaining after the treatment with each drug independently. CDI < 1 indicates a synergistic effect, CDI = 1 indicates an additive effect, and CDI > 1 indicates antagonism. CDI < 0.7 indicates that the drugs are significantly synergistic.
